# Aggregated occurrence records of the federally endangered Poweshiek skipperling (*Oarisma poweshiek*)

**DOI:** 10.3897/BDJ.6.e29081

**Published:** 2018-09-27

**Authors:** Michael W Belitz, Lillian K Hendrick, Michael J Monfils, David L Cuthrell, Christopher J Marshall, Akito Y Kawahara, Neil S Cobb, Jennifer M Zaspel, Andrew M Horton, Stacey L Huber, Andrew D Warren, Grace A Forthaus, Anna K Monfils

**Affiliations:** 1 Central Michigan University and Institute for Great Lakes Research, Mount Pleasant, United States of America Central Michigan University and Institute for Great Lakes Research Mount Pleasant United States of America; 2 Michigan Natural Features Inventory, Michigan State University Extension, Lansing, United States of America Michigan Natural Features Inventory, Michigan State University Extension Lansing United States of America; 3 Oregon State Arthropod Collection, Department of Integrative Biology, Corvallis, United States of America Oregon State Arthropod Collection, Department of Integrative Biology Corvallis United States of America; 4 McGuire Center for Lepidoptera and Biodiversity, Florida Museum of Natural History, University of Florida, Gainesville, United States of America McGuire Center for Lepidoptera and Biodiversity, Florida Museum of Natural History, University of Florida Gainesville United States of America; 5 Merriam-Powell Center for Environmental Research and Department of Biological Sciences, Northern Arizona University, Flagstaff, United States of America Merriam-Powell Center for Environmental Research and Department of Biological Sciences, Northern Arizona University Flagstaff United States of America; 6 Department of Zoology, Milwaukee Public Museum, Milwaukee, United States of America Department of Zoology, Milwaukee Public Museum Milwaukee United States of America; 7 Minnesota-Wisconsin Ecological Services Field Office, U.S. Fish and Wildlife Service, Bloomington, United States of America Minnesota-Wisconsin Ecological Services Field Office, U.S. Fish and Wildlife Service Bloomington United States of America

**Keywords:** Butterfly Conservation, Distribution, Endangered Species, Hesperiidae, Location, Occurrence, *Oarisma poweshiek*

## Abstract

**Background:**

Primary biodiversity data records that are open access and available in a standardised format are essential for conservation planning and research on policy-relevant time-scales. We created a dataset to document all known occurrence data for the Federally Endangered Poweshiek skipperling butterfly [*Oarisma poweshiek* (Parker, 1870; Lepidoptera: Hesperiidae)]. The Poweshiek skipperling was a historically common species in prairie systems across the upper Midwest, United States and Manitoba, Canada. Rapid declines have reduced the number of verified extant sites to six. Aggregating and curating Poweshiek skipperling occurrence records documents and preserves all known distributional data, which can be used to address questions related to Poweshiek skipperling conservation, ecology and biogeography. Over 3500 occurrence records were aggregated over a temporal coverage from 1872 to present. Occurrence records were obtained from 37 data providers in the conservation and natural history collection community using both “HumanObservation” and “PreservedSpecimen” as an acceptable basisOfRecord. Data were obtained in different formats and with differing degrees of quality control. During the data aggregation and cleaning process, we transcribed specimen label data, georeferenced occurrences, adopted a controlled vocabulary, removed duplicates and standardised formatting. We examined the dataset for inconsistencies with known Poweshiek skipperling biogeography and phenology and we verified or removed inconsistencies by working with the original data providers. In total, 12 occurrence records were removed because we identified them to be the western congener *Oarisma garita* (Reakirt, 1866). This resulting dataset enhances the permanency of Poweshiek skipperling occurrence data in a standardised format.

**New information:**

This is a validated and comprehensive dataset of occurrence records for the Poweshiek skipperling (*Oarisma poweshiek*) utilising both observation and specimen-based records. Occurrence data are preserved and available for continued research and conservation projects using standardised Darwin Core formatting where possible. Prior to this project, much of these occurrence records were not mobilised and were being stored in individual institutional databases, researcher datasets and personal records. This dataset aggregates presence data from state conservation agencies, natural heritage programmes, natural history collections, citizen scientists, researchers and the U.S. Fish & Wildlife Service. The data include opportunistic observations and collections, research vouchers, observations collected for population monitoring and observations collected using standardised research methodologies. The aggregated occurrence records underwent cleaning efforts that improved data interoperablitity, removed transcription errors and verified or removed uncertain data. This dataset enhances available information on the spatiotemporal distribution of this Federally Endangered species. As part of this aggregation process, we discovered and verified Poweshiek skipperling occurrence records from two previously unknown states, Nebraska and Ohio.

## Introduction

The Poweshiek skipperling [*Oarisma poweshiek* (Parker, 1870) (Lepidoptera: Hesperiidae)] is a small-bodied (approximately 2.3 – 3.0 cm), univoltine butterfly that was listed in 2014 as Federally Endangered in both the United States and Canada (COSEWIC - Committee on the Status of Endangered Wildlife in Canada 2014, USFWS - U.S. Fish and Wildlife Service 2014). As recently as the mid-1990s, Poweshiek skipperling were widespread and reliably observed in prairie systems of western Minnesota and eastern South Dakota (Schlicht et al. 2008), but in the past two decades, a dramatic range-wide reduction in populations has occurred (Swengel et al. 2010, Pogue et al. 2016). The Poweshiek skipperling is now known only from approximately 1% of the sites where it once occurred (Marquardt et al. 2018).

Historically, the core range of Poweshiek skipperling was in prairies of western Minnesota and eastern South Dakota (Selby 2005, Saarinen et al. 2016). Currently, there are six extant populations of Poweshiek skipperling known that occur on the margins of its historic range. Four populations occur in prairie fens in Michigan, USA, one in a mesic prairie in Wisconsin, USA and one in a tallgrass prairie system in Manitoba, Canada (Delphey et al. 2016). Although conservation initiatives focused on captive rearing and habitat management are underway (Delphey et al. 2016), limited information on the biology and biogeography of the Poweshiek skipperling is available, possibly further restricting the current success of these projects.

Primary biodiversity data are critical in driving conservation management of endangered species and ecosystems (Hardisty et al. 2013). Refined, validated and reformatted spatiotemporal distribution data can provide information for research and management projects related to the conservation and ecology of the Poweshiek skipperling. Our goal was to leverage the collected knowledge and expertise of the natural history collection and conservation community to aggregate a comprehensive and validated dataset of Poweshiek skipperling occurrence records. Aggregating, cleaning and verifying occurrences inclusive of both human observations and preserved specimens naturally promoted interdisciplinary collaboration between project partners. Mobilising the collective knowledge and expertise of interdisciplinary groups can broaden the effect of research by addressing the complexities and challenges related to biodiversity decline (Marquardt et al. 2018). Here, we compile occurrence records from both human observations and preserved specimens that have undergone a comprehensive cleaning process, providing accessible and curated data.

## General description

### Purpose

Poweshiek skipperling face a high risk of extinction (COSEWIC - Committee on the Status of Endangered Wildlife in Canada 2014, USFWS - U.S. Fish and Wildlife Service 2014), making data discovery, aggregation and sharing an urgent and valuable endeavour. We aggregated and curated occurrence records of the Federally Endangered Poweshiek skipperling to examine and validate the distribution of this species. To this degree, the data are being used in developing ecological niche models to examine the correlation between climate and land use variables and the presence of Poweshiek skipperling through space and time (Belitz et al. unpublished data). The publication of occurrence records will provide information and encourage continued research into the biology and conservation of Poweshiek skipperling, while also preserving aggregated data in a standardised format that has undergone a cleaning process.

## Project description

### Title

Aggregated occurrence records of the Federally Endangered Poweshiek skipperling (*Oarisma poweshiek*)

### Study area description

The study area covered all sites within the historic range of Poweshiek skipperling, including ten states (Illinois, Indiana, Iowa, Michigan, Minnesota, Nebraska, North Dakota, South Dakota, Ohio and Wisconsin) in the Midwest, United States and southeast Manitoba, Canada.

## Sampling methods

### Sampling description

Poweshiek skipperling occurrence records were aggregated from the following sources: federal agencies (e.g. U.S. Fish & Wildlife Service), natural heritage member programmes (e.g. Michigan Natural Features Inventory), state conservation agencies (e.g. Minnesota DNR, South Dakota GFP), citizen scientists (e.g. iNaturalist, The Lepidopterists’ Society) and natural history collections (Table 1). Both “HumanObservation” and “PreservedSpecimen” were included as occurrence records. Occurrence records were also gathered from the following data aggregators: Global Biodiversity Information Facility (GBIF) and Lepidoptera of North America Network (LepNet). LepNet is a thematic collection network (TCN), whose data included human observations from citizen scientists (via iNaturalist and The Lepidopterists' Society) and preserved specimens from natural history collections (Seltmann et al. 2017). Many records from LepNet were uploaded in response to requests that we sent to the collections' community (Shepard and Marshall 2017). Prior to our study, there were seven Poweshiek skipperling records in the LepNet repository. As of July 2018, there were 776 records. LepNet also assisted in procuring data from regional collections whose data were not available through publicly accessible repositories. Data from regional collections and smaller projects can enhance scientific inquiry and statistical modelling (Glon et al. 2017, Heidorn 2008). We accessed these data sources by transcribing and standardising specimen metadata that we gathered by transcribing metadata at the physical collection or by curating metadata that was sent in a variety of spreadsheet, text files and word document formats. A part of our data aggregation effort mobilised citizen scientists through a Notes from Nature expedition, where citizen scientists transcribed specimen label data (Hill et al. 2012). Aggregated data included research vouchers, opportunistic observations and collections, observations collected for population monitoring (Selby 2005, Swengel et al. 2010) and observations collected using standardised research methodologies (Pogue et al. 2016).

Occurrence data, lacking associated geographical coordinates, were georeferenced using GEOLocate (Rios and Bart 2010). Records with TRS (Township, Range and Section) data were georeferenced using the Bureau of Land Management (BLM) single point translation using Earthpoint (www.earthpoint.us/Townships.aspx). If geographic coordinates were not originally provided in decimal degrees, they were converted to decimal degrees, datum WGS84.

### Quality control

In the process of vetting the dataset, we identified records that appeared to be outside the range of the Poweshiek skipperling. Images of specimens georeferenced outside the previously known range of the Poweshiek skipperling were obtained and checked by DL Cuthrell, who has worked with this species for over 20 years, to ensure the correct identification of the specimen. Specimens collected in Montana, Colorado, Western Nebraska and Western Manitoba were misidentified as *O. poweshiek* and instead were *O. garita.* However, five specimens collected from Nebraska and one collected in Ohio were confirmed as *O. poweshiek*, expanding the known states that once had Poweshiek skipperling. Using our collective knowledge of historic Poweshiek skipperling sites and our aggregated dataset, we were able to check and refine georeferenced occurrence records. Geographic coordinates of occurrence records that were incorrectly georeferenced were changed to represent coordinates consistent with the locality listed in the occurrence metadata. We mask the locality information of the six extant Poweshiek skipperling sites to protect the Federally Endangered species and its vulnerable prairie habitat.

basisOfRecord: Data records with an unknown basisOfRecord were removed from our dataset to ensure the specific nature of the data record was documented.

scientificName: The Poweshiek skipperling was originally described as *Hesperia powesheik* by Parker (1870) and numerous occurrence records were listed as *Oarisma powesheik*. The butterfly's type series includes 33 specimens collected in Poweshiek County, Iowa. We aggregated occurrence records listed as *O. poweshiek*, *O. powesheik* and *H. poweshiek* and chose to standardise all taxonomic names to reflect the accepted spelling, *Oarisma poweshiek* (Parker, 1870) as printed in Pelham (2008).

eventDate: We contacted original data providers to check label transcription and identification of Poweshiek skipperling occurrences that were listed outside the expected Poweshiek skipperling flight period of mid-June to mid-July. We removed eventDates, that were automatically filled with an institution’s default date (e.g. 1700-01-01). Any cells in the dataset that were filled with N/A abbreviations were removed.

Data within columns were edited to adopt a controlled vocabulary and the Darwin Core standards were used when applicable. Original data were retained when controlled vocabulary could not be utilised. Spelling errors or errors in transcription were noted and changed to reflect correct spelling. We removed any duplicate records that were gathered from multiple sources by removing occurrences with duplicate occurrenceID and/or catalogNumber. Original data were received and downloaded with varying degrees of indexing. Cleaned data were formatted according to Darwin Core standards (Wieczorek et al. 2012) and primary data providers were informed of any edits.

## Geographic coverage

### Description

**Figure 1.**
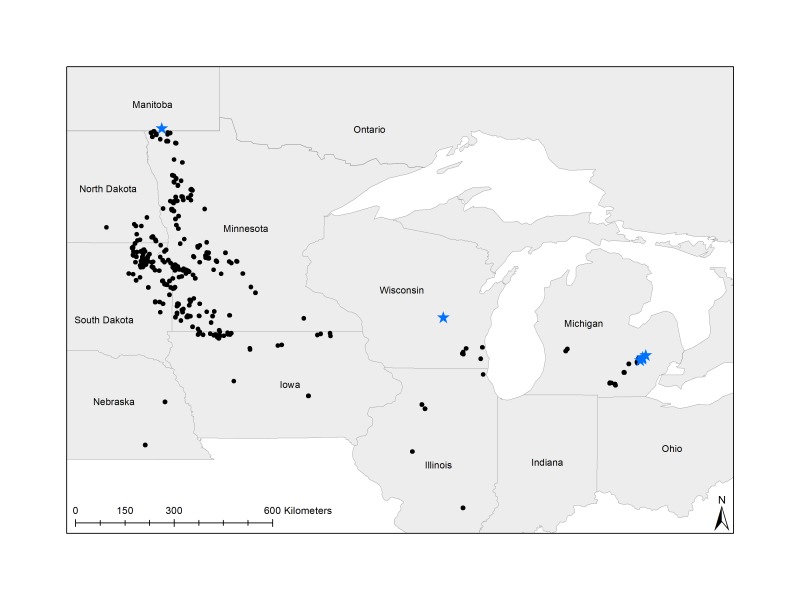
Distribution of Poweshiek skipperling occurrence records. Occurrence records that are georeferenced at a state centroid resolution are not shown. Stars display the six extant sites (four occur in eastern Michigan, one in Wisconsin and one in Manitoba).

The geographic range of the dataset covers nine U.S. states (North Dakota, South Dakota, Nebraska, Minnesota, Iowa, Wisconsin, Illinois, Michigan and Ohio) and one Canadian province (Manitoba; Fig. 1). The state with the greatest number of Poweshiek skipperling occurrence records was Michigan (Table 2).

### Coordinates

38.669 and 49.133 Latitude; -98.253 and -83.468 Longitude.

## Taxonomic coverage

### Description

This dataset is devoted to one species of Lepidoptera in the family Hesperiidae. The species is *Oarisma poweshiek* (Parker, 1870).

### Taxa included

**Table:** 

Rank	Scientific Name	Common Name
kingdom	Animalia	Animals
phylum	Arthropoda	Arthropods
class	Insecta	Insects
order	Lepidoptera	Butterflies and Moths
family	Hesperiidae	Skippers
species	*Oarisma poweshiek*	Poweshiek skipperling

## Temporal coverage

### Notes

**Figure 2.**
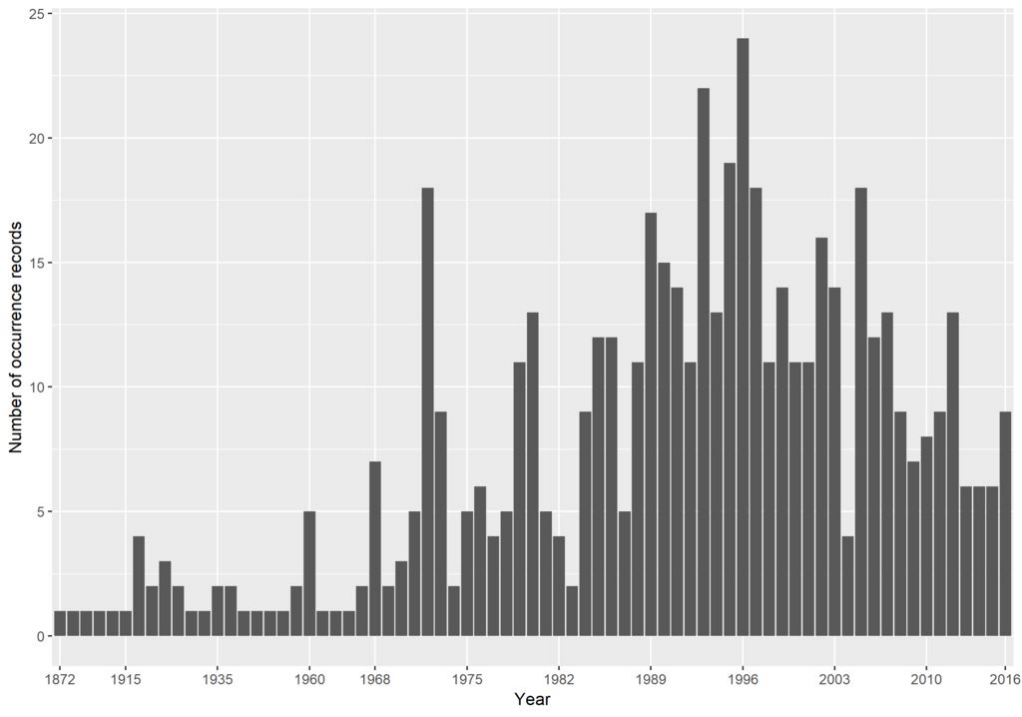
Temporal profile of the number of Poweshiek skipperling occurrences that were documented on unique days. Survey effort for this species increased in the mid-1990s (Selby 2005, Swengel et al. 2010). Poweshiek skipperling were listed as federally endangered in the United States and Canada in 2014 (COSEWIC - Committee on the Status of Endangered Wildlife in Canada 2014, USFWS - U.S. Fish and Wildlife Service 2014).

1872 – present (Fig. 2). Poweshiek skipperling were originally described by Parker (1870) based on 33 individuals collected in Grinnell, Iowa. Occurrence records of Poweshiek skipperling specimen collected by HW Parker in Grinnell, Iowa are aggregated in our dataset but do not have an associated eventDate.

## Usage rights

### Use license

Other

### IP rights notes

See individual records for usage rights.

## Data resources

### Data package title

Aggregated occurrence records of the federally endangered Poweshiek skipperling (*Oarisma poweshiek*)

### Number of data sets

1

### Data set 1.

#### Data set name

Oarisma poweshiek occurrences

#### Data format

Darwin Core Archive

#### Number of columns

49

#### Download URL

http://ipt.idigbio.org/resource?r=cmc

#### Data format version

1.8

#### Description

Data are formatted according to Darwin Core standards (http://rs.tdwg.org/dwc/terms) and the column labels and column descriptions are based on this standard.

**Data set 1.** 

Column label	Column description
institutionCode	The name or acronym in use by the institution having custody of the object(s) or information referred to in the record.
collectionCode	The name, acronym, coden or initialism identifying the collection or dataset from which the record was derived.
basisOfRecord	The specific nature of the data record. We used a Darwin Core controlled vocabulary for our basisOfRecord that included "PreservedSpecimen" and "HumanObservation".
occurrenceID	An identifier for the Occurrence (as opposed to a particular digital record of the occurrence). In the absence of a persistent global unique identifier, construct one from a combination of identifiers in the record that will most closely make the occurrence ID globally unique. In this dataset, occurrence records use the ID number from its holding facility when applicable. Occurrence records that did not have a unique ID were given their own unique observation ID.
catalogNumber	An identifier for the record within the data set or collection.
otherCatalogNumbers	A list of previous or alternative fully qualified catalogue numbers of the catalogued item whether in the current collection or in any other.
scientificName	The full scientific name.
scientificNameAuthorship	The authorship information for the scientificName formatted according to the conventions of the applicable nomenclaturalCode.
genus	The full scientific name of the genus in which the taxon is classified.
specificEpithet	The name of the first or species epithet of the scientificName.
identifiedBy	A list of names of people, groups or organisations who assigned the taxon to the subject.
dateIdentified	The date-time in the Common Era calendar in which the object or observation was identified as being a member of the taxon given in the scientificName.
recordedBy	A list of names of people, groups or organisations responsible for recording the original Occurrence. The primary collector or observer.
eventDate	The date-time or interval during which an Event occurred. For occurrences, this is the data-time when the event was recorded.
year	The four-digit year in which the Event occurred, according to the Common Era Calendar.
day	The integer day of the month on which the Event occurred.
month	The ordinal month in which the Event occurred.
verbatimEventDate	The verbatim original representation of the date and time information for an Event.
habitat	A category or description of the habitat in which the Event occurred.
lifeStage	Indicates the life stage present.
sex	The sex of the individual represented.
individualCount	The number of individuals represented present at the time of the Occurrence.
samplingProtocol	The name of, reference to or description of the method or protocol used during an Event.
samplingEffort	The amount of effort expended during an Event.
preparations	A list of preparations and preservation methods for a specimen.
country	The name of the country or major administrative unit in which the Location occurs. We used the recommended best practice to use the Getty Thesaurus of Geographic Names as the controlled vocabulary.
stateProvince	The name of the next smaller administrative region than country (state, province, canton, department, region etc.) in which the Location occurs.
county	The full, unabbreviated name of the next smaller administrative region than stateProvince (county, shire, department etc.) in which the Location occurs.
municipality	The full, unabbreviated name of the next smaller administrative region than county (city, municipality etc.) in which the Location occurs.
locality	The specific description of the place. Less specific geographic information can be provided in other geographic terms (higherGeography, continent, country, stateProvince, county, municipality, waterBody, island, islandGroup). This term may contain information modified from the original to correct perceived errors or to standardise the description.
locationRemarks	Comments or notes about the Location.
decimalLatitude	The latitude of the location from which the catalogued item was collected, expressed in decimal degrees.
decimalLongitude	The longitude of the location from which the catalogued item was collected, expressed in decimal degrees.
geodeticDatum	The ellipsoid, geodetic datum or spatial reference system (SRS) upon which the geographic coordinates given in decimalLatitude and decimalLongitude are based. Recommended best practice is use of the EPSG code as a controlled vocabulary to provide an SRS, if unknown. Otherwise use of a controlled vocabulary for the name or code of the geodetic datum, if unknown.
coordinateUncertaintyInMeters	The horizontal distance (in metres) from the given decimalLatitude and decimalLongitude describing the smallest circle containing the whole of the Location. Leave the value empty if the uncertainty is unknown, cannot be estimated or is not applicable (because there are no coordinates). Zero is not a valid value for this term.
verbatimCoordinates	The verbatim original spatial coordinates of the Location. The coordinate ellipsoid, geodeticDatum or full Spatial Reference System (SRS) for these coordinates should be stored in verbatimSRS.
georeferencedBy	A list (concatenated and separated) of names of people, groups or organisations who determined the georeference (spatial representation) for the Location.
georeferenceProtocol	A description or reference to the methods used to determine the spatial footprint, coordinates and uncertainties.
georeferenceSources	A list (concatenated and separated) of maps, gazetteers or other resources used to georeference the Location, described specifically enough to allow anyone in the future to use the same resources.
georeferenceRemarks	Notes or comments about the spatial description determination, explaining assumptions made in addition or opposition to those formalised in the method referred to in georeferenceProtocol.
modified	The most recent data-time on which the resource was changed.
rightsHolder	A person or organisation owning or managing rights over the resource.
license	A legal document giving official permission to do something with the resource.
references	A related resource that is referenced, cited or otherwise pointed to by the described resource.
bibliographicCitation	A bibliographic reference for the resource as a statement indicating how this record should be cited (attributed) when used. Any data records that were edited cite this data paper in this column.
ownerInstitutionCode	The name (or acronym) in use by the institution having ownership of the object(s) or infomation referred to in the record.
occurrenceRemarks	Comments or notes about the occurrence.
informationWithheld	Additional information that exists, but that has not been shared in the given record. In this dataset, we withhold information regarding location of extant sites and locality information from specific agencies.
eventTime	The time or interval during which an Event occurred. Time is listed in time zone of the respective occurrence record.

## Figures and Tables

**Table 1.** Source of Poweshiek skipperling occurrence records. The total number of occurrences (3676) obtained from each source are listed as of July 2018.

**Source**	**Total Occurrences**
BugGuide (LepNet)	4
Cal Academy of Sciences	68
Canadian National Collection	2
Cleveland Museum of Natural History Invertebrate Zoology (InvertEBase)	6
Chicago Academy of Sciences - Peggy Notebaert Nature Museum	1
Colorado State University - C.P. Gillette Museum of Arthropod Diversity (LepNet)	22
Denver Museum of Nature & Science - (LepNet)	2
Drexel University - Academy of Natural Sciences (LepNet)	6
Field Museum of Natural History	5
Florida Museum of Natural History (LepNet)	155
Georgia Museum of Natural History – University of Georgia Collection of Arthropods (LepNet)	1
Harvard University - Museum of Comparative Zoology (LepNet)	11
Illinois Natural History Survey	61
iNaturalist (LepNet)	1
Manitoba Conservation Data Centre	2
Michigan Natural Features Inventory	1790
Michigan State University - Albert J. Cook Arthropod Research Collection (LepNet)	48
Milwaukee Public Museum (LepNet)	66
Minnesota Department of Natural Resources	180
Minnesota Natural Heritage Program	11
Mississippi State University - Mississippi Entomological Museum (LepNet)	12
National Museum of Natural History	82
North Dakota State University	31
Oregon State University - Arthropod Collection (LepNet)	14
South Dakota Department of Game, Fish, and Parks	39
South Dakota Natural Heritage Program	1
South Dakota State University	36
Texas A&M University - Biodiversity Research and Teaching Collection	33
The Lepidopterists' Society (LepNet)	28
The Manitoba Museum (LepNet)	206
The Ohio State University - C.A. Triplehorn Insect Collection (LepNet)	65
U.S. Fish and Wildlife Service	549
UC Davis - Bohart Museum of Entomology (LepNet)	2
University of California Berkeley - Essig Museum of Entomology Collection (LepNet)	2
University of Minnesota - Insect Collection (LepNet)	113
University of Utah - Natural History Museum of Utah (LepNet)	4
Yale University - Peabody Museum (LepNet)	17

**Table 2.** The number of Poweshiek skipperling occurrence records across the study area as of July 2018.

**Country**	**State/Province**	**Total**
United States	Illinois	10
United States	Iowa	352
United States	Michigan	2043
United States	Minnesota	624
United States	Nebraska	5
United States	North Dakota	56
United States	Ohio	1
United States	South Dakota	238
United States	Wisconsin	96
Canada	Manitoba	228
